# Efficacy of photodynamic therapy with various photosensitizers for peri-implantitis treatment: a systematic review and meta-analysis of randomized clinical trials

**DOI:** 10.1007/s10103-025-04612-7

**Published:** 2025-09-16

**Authors:** Xin Li, Hang Liu, Lian Yang, Yiming Ji, Dingli Feng, Ruiqi Shao, Guanhua Zhang, Shichen Lin, Shaoyu Duan, Xue Wu

**Affiliations:** Department of Stomatology, Electric Power Teaching Hospital, Capital Medical University, Beijing, China

**Keywords:** Peri-implantitis, Photodynamic therapy, Toluidine blue, Photosensitizers, Meta-analysis, Clinical outcomes, Implant dentistry

## Abstract

To systematically evaluate the efficacy of photodynamic therapy in treating peri-implantitis and compare the effectiveness of various photosensitizers based on randomized clinical trials. A systematic review and meta-analysis were performed following Preferred Reporting Items for Systematic Reviews and Meta-Analyses (PRISMA) guidelines, incorporating data from PubMed/MEDLINE, Embase, Web of Science, and CNKI up to December 31, 2024. Randomized clinical trials that assessed the efficacy of photodynamic therapy with various photosensitizers for peri-implantitis were included. Primary outcomes included bleeding on probing, probing depth, plaque index, clinical attachment level, crestal bone loss, and bleeding index. Standardized mean differences (SMD) with 95% confidence intervals (CIs) were calculated for each outcome to quantify the effect size. Risk of bias was assessed using the Cochrane risk-of-bias tool. 13 randomized clinical trials involving 678 participants were included in the analysis. Photodynamic therapy significantly improved several clinical outcomes compared to controls: bleeding on probing (SMD = -0.49, 95% CI: -0.89 to -0.09), probing depth (SMD = -1.49, 95% CI: -2.31 to -0.66), plaque index (SMD = -1.11, 95% CI: -1.98 to -0.25), and crestal bone loss (SMD = -0.53, 95% CI: -0.89 to -0.17). Subgroup analyses revealed that Toluidine Blue was the most effective photosensitizer, showing superior improvements across multiple outcomes. Photodynamic therapy is an effective treatment for peri-implantitis, particularly when using Toluidine Blue, which consistently outperformed other photosensitizers in improving clinical outcomes. These findings provide strong evidence for integrating photodynamic therapy into peri-implantitis management protocols, offering a promising, minimally invasive alternative to conventional treatments. Future studies should focus on optimizing photodynamic therapy protocols, assessing long-term outcomes, and evaluating its effectiveness in diverse patient populations.

Clinical trial number: PROSPERO (CRD42024600326).

**Supplementary Information:**

The online version contains supplementary material available at 10.1007/s10103-025-04612-7.

## Introduction

Photodynamic Therapy (PDT), first introduced in the early 20th century and later established in oncology for the treatment of skin and esophageal cancers, is a non-invasive therapeutic technique that combines a light-sensitive photosensitizer with a specific wavelength of light to generate reactive oxygen species (ROS) [1]. These ROS exert cytotoxic effects on target cells and are particularly effective against microbial biofilms. Over the past two decades, PDT has gained traction in various medical disciplines—including dermatology, ophthalmology, and infectious disease management—owing to its antimicrobial efficacy and minimal side effects [2]. In dentistry, PDT has emerged as a promising adjunctive treatment for periodontitis and peri-implant infections, especially in cases resistant to conventional mechanical or chemical interventions [3].

Peri-implantitis is one of the principal complications compromising the long-term success of dental implants, characterized by an inflammatory process that leads to progressive bone loss around the implant [4]. This condition can result in both functional impairment and patient dissatisfaction, often manifesting as bleeding on probing, increased probing depth, and eventual implant instability. Despite the overall success rates of dental implants, peri-implantitis remains a significant clinical concern, with its prevalence varying widely (ranging from 5% to over 40% in some populations) due to differences in diagnostic criteria, patient risk profiles, and follow-up periods [5]. Although multiple factors contribute to peri-implantitis—including microbial infection, host immune response, and implant surface characteristics—persistent biofilm accumulation on the implant surface is widely recognized as a major etiological agent [6]. Importantly, the multifactorial pathogenesis of peri-implantitis has rendered conventional treatments, such as mechanical debridement and local or systemic antimicrobials, variably successful and sometimes insufficient for long-term disease control. Against this backdrop, there is a pressing need for therapies that can reliably disrupt pathogenic biofilms and reduce inflammation without causing excessive damage to peri-implant tissues.

Mechanical debridement, chemical therapy (e.g., chlorhexidine), and surgical intervention constitute the primary strategies in peri-implantitis management. However, these modalities may result in incomplete biofilm removal, potential damage to implant surfaces, adverse tissue reactions, and extended recovery periods for patients [6–8]. Consequently, there is a recognized need for minimally invasive and highly targeted alternatives capable of overcoming these clinical limitations. Photodynamic Therapy (PDT) has attracted considerable attention as a complementary or alternative approach due to its ability to selectively target pathogenic microorganisms while minimizing collateral tissue damage [9]. When a photosensitizer is activated by light of a specific wavelength, reactive oxygen species (ROS) are generated to disrupt microbial cell walls and reduce inflammation [10].

Nonetheless, a key aspect of PDT for peri-implantitis management lies in the choice of photosensitizer, which can markedly affect therapeutic outcomes. Toluidine Blue, Phenothiazine chloride, and Methylene Blue, for instance, exhibit distinct bactericidal mechanisms, penetration capabilities, and spectral absorption properties, leading to variations in clinical efficacy and sometimes conflicting evidence as to which agent offers the most substantial therapeutic advantage [3, 11]. Multiple randomized controlled trials (RCTs) have also reported heterogeneous results, potentially attributable to differences in photosensitizer concentrations, laser parameters, follow-up durations, and study designs [12–14].This divergence underscores the urgent need to determine the optimal photosensitizer for PDT—particularly given the increasing prevalence of peri-implantitis and the therapeutic limitations of mechanical or chemical modalities alone. Such methodological and clinical inconsistencies make it difficult to draw definitive conclusions about the most effective photosensitizer or the ideal PDT regimen for peri-implantitis. As these unresolved issues continue to challenge both researchers and clinicians, a comprehensive meta-analysis is warranted to synthesize current evidence, elucidate best practices, and refine treatment guidelines for this increasingly prevalent condition.

To address these inconsistencies and provide a clearer understanding of the role of photodynamic therapy, we conducted a comprehensive systematic review and meta-analysis of randomized clinical trials evaluating its use in peri-implantitis management. The primary objective of this study was twofold: first, to evaluate the overall efficacy of photodynamic therapy in improving clinical and radiographic outcomes; and second, to compare the relative effectiveness of different photosensitizers through subgroup analyses. The findings of this analysis will help refine peri-implantitis treatment strategies, advance the use of PDT in clinical practice, and guide subsequent studies toward optimizing long-term outcomes through photosensitizer selection and standardized application methods.

## Methods

This pre-registered systematic review and meta-analysis was conducted following the Preferred Reporting Items for Systematic Reviews and Meta-Analyses (PRISMA) guidelines [15].The protocol has been registered in PROSPERO (International Prospective Register of Systematic Reviews) a priori under registration number CRD42024600326.

### PICO framework and focused research question

To ensure a structured and transparent approach to study selection, this systematic review and meta-analysis adhered to the PICO (Population, Intervention, Comparison, Outcome) framework, structured as follows:

- Population (P): Adults (≥ 18 years) diagnosed with peri-implant diseases, including peri-implant mucositis and peri-implantitis.
- Intervention (I): PDT as a primary treatment modality, with different photosensitizers (Toluidine Blue, Phenothiazine chloride, and Methylene Blue) serving as subgroup variables rather than independent interventions. PDT was applied using laser technologies such as diode, argon, or Nd: YAG lasers.
- Comparison (C): Conventional non-surgical interventions, including mechanical debridement and chemical treatment (e.g., chlorhexidine), with or without laser technology.
- Outcome (O): Clinical and radiographic indicators of peri-implant health, including bleeding on probing (BOP), probing depth (PD), plaque index (PI), clinical attachment level (CAL), crestal bone loss (CBL), and bleeding index (BI).

Although the PROSPERO protocol focused on the overall efficacy of photodynamic therapy, it did not pre-specify subgroup analyses based on photosensitizer type. However, during full-text screening and data extraction, we identified variations in photosensitizer use across studies. To address the resulting heterogeneity and improve interpretability, subgroup analyses were subsequently conducted based on photosensitizer type. Based on this framework, the focused research question guiding this study was: “What is the efficacy of PDT compared to conventional non-surgical interventions in the treatment of peri-implantitis, and how do different photosensitizers influence the clinical effectiveness of PDT?”

### Search strategy

A comprehensive literature search was conducted across multiple electronic databases, including PubMed/MEDLINE, Embase, Web of Science, and the China National Knowledge Infrastructure (CNKI), covering all available records up to December 31, 2024. No language restrictions were applied during the search process, and non-English studies were translated when necessary to ensure eligibility assessment and data extraction.

The search strategy combined controlled vocabulary (e.g., MeSH terms in PubMed/MEDLINE and Emtree in Embase) and free-text terms tailored to each database’s indexing system. Although the core search concepts remained consistent across databases, the syntax, field codes, and operators were adapted accordingly to maximize retrieval sensitivity and specificity. Detailed, database-specific search strings—including Boolean operators and field tags—are provided in Supplementary File 1.

Additionally, relevant articles and reviews’ reference lists were manually searched to identify any studies potentially missed during the electronic search. Title and abstract screening, as well as full-text review, were independently conducted by two researchers (L.Y. and H.L.), with any disagreements resolved through discussion or consultation with a third reviewer (X.L.). Inter-rater agreement for study screening was substantial, with a Cohen’s Kappa coefficient of 0.81.

### Inclusion and exclusion criteria

Studies eligible for inclusion in this systematic review and meta-analysis met the following criteria: (1) participants were adults (≥ 18 years) diagnosed with peri-implant diseases, including peri-implant mucositis and peri-implantitis; (2) the intervention group received PDT using various photosensitizers with laser systems commonly employed in contemporary clinical protocols (e.g., diode, argon, or Nd: YAG lasers); (3) the control group underwent mechanical debridement, chemical treatment (e.g., chlorhexidine), or non-surgical interventions without laser technology; (4) outcomes were reported for at least one of the following: clinical attachment loss (CAL), probing depth (PD) or bleeding on probing (BOP); (5) only randomized controlled trials (RCTs) with a minimum follow-up of 3 months were included. Exclusion criteria included: reviews, case reports, case series, conference abstracts, technical reports, studies with incomplete or duplicated data, and studies that did not specify the type of laser technology used or those providing incomplete or non-extractable outcome data.

Data extraction was independently performed by two reviewers (L.Y. and H.L.), with any discrepancies resolved by consensus or consultation with a third reviewer (X.L.). The inter-rater agreement for data extraction was high (Cohen’s Kappa = 0.84). Extracted data included study characteristics (author, year, country), participant demographics (age, sex), disease classification, PDT parameters (photosensitizer type, laser wavelength, power), and clinical outcomes (CAL, PD, BOP, PI). Where data were incomplete, corresponding authors were contacted up to four times over six weeks to request additional information.

### Outcomes

The primary outcome measures of interest included BOP, PD, PI, CAL, CB, and BI. These outcomes were selected to comprehensively assess both clinical and radiographic indicators of peri-implantitis treatment efficacy, providing a robust evaluation of the impact of PDT on peri-implant tissue health.

### Risk of bias assessment

Risk of bias was assessed using the revised Cochrane risk-of-bias tool for randomized trials (RoB 2) [16], which evaluates the randomization process, deviations from intended interventions, missing outcome data, outcome measurement, and selective reporting. Any disagreements between reviewers (Y.J. and D.F.) were resolved through discussion, with a third reviewer (X.L.). involved when necessary to ensure an unbiased and rigorous evaluation. The inter-rater agreement for risk of bias assessment was acceptable (Cohen’s Kappa = 0.79).

### Data synthesis

Data synthesis was performed using Stata software (version 14, StataCorp LLC, Texas, USA). The heterogeneity across studies was assessed using the I^2^ statistic, with thresholds of I^2^ < 25% indicating low heterogeneity, 25%−50% indicating moderate heterogeneity, and > 50% indicating high heterogeneity [17]. A fixed-effects model was used when I^2^ ≤ 25%, while a random-effects model was applied when I^2^ > 25% to account for variability across studies. For continuous outcomes, standardized mean differences (SMD) with 95% confidence intervals (CIs) were calculated, adjusting for differences in outcome measurement methods and units.

A sensitivity analysis was conducted by sequentially excluding studies to evaluate the impact of individual studies on the overall effect size and heterogeneity. If significant variability was identified, the excluded studies were re-examined based on the inclusion criteria and study quality to assess their potential impact on the results. Potential publication bias was evaluated using funnel plots and Egger’s test [18], with a p-value < 0.05 indicating the presence of bias.

## Results

### Characteristics of included studies

**Fig. 1:**
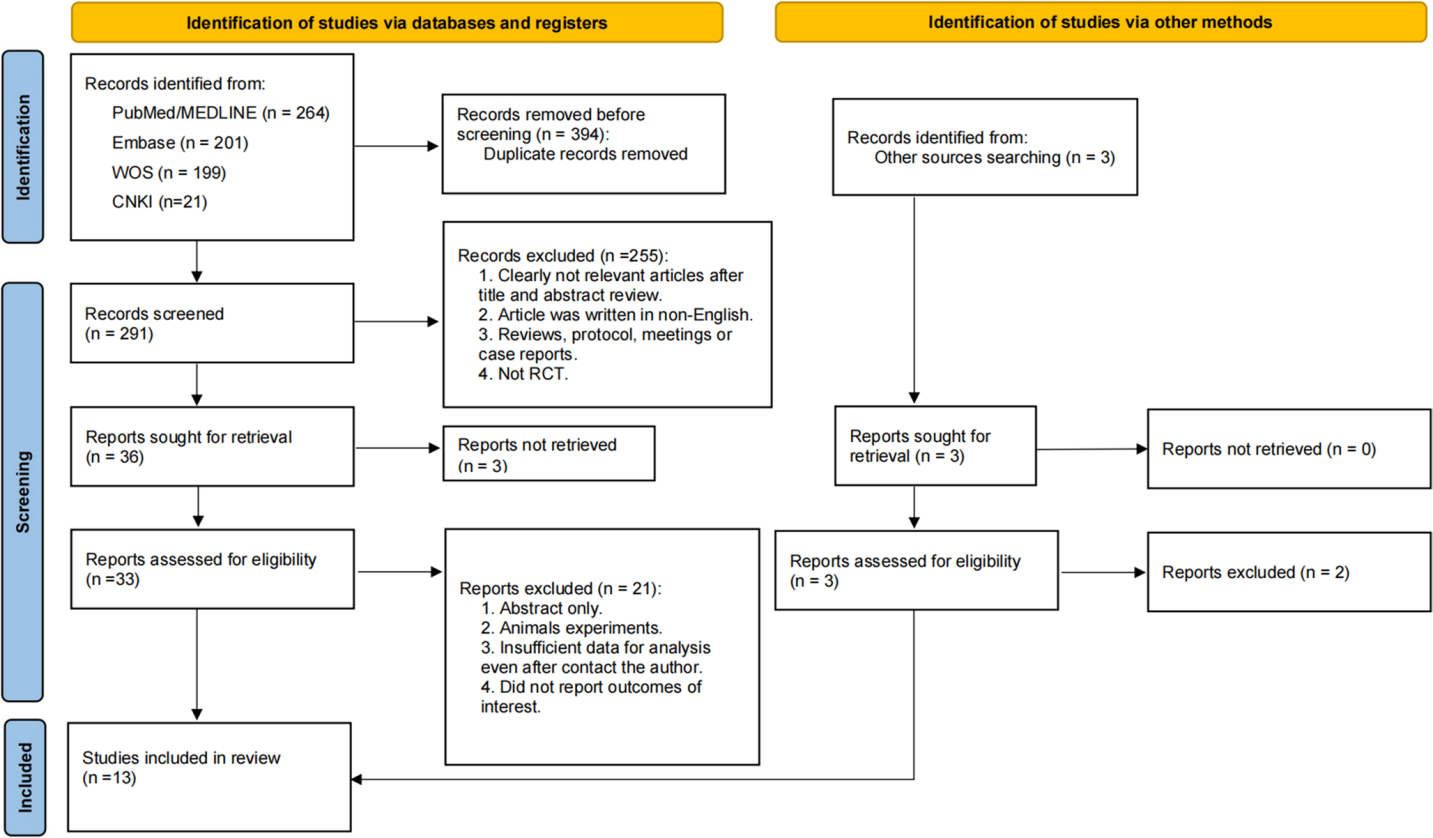
PRISMA Flow diagram of the search process for studies

The initial electronic search identified a total of 685 records. After removing duplicates, 291 records were screened based on titles and abstracts, resulting in the full-text assessment of 36 articles. Of these, thirteen studies met the predefined inclusion criteria and were subsequently included in the meta-analysis (Fig. 1), comprising a total of 678 participants [13, 14, 19–29]. The sample sizes of the included studies ranged from 20 to 131 participants, with a median sample size of 40. The participants’ ages spanned from 33.6 to 65.2 years, with a median age of 51.1 years. Among the included studies, six used Phenothiazine chloride as the photosensitizer, four utilized Toluidine Blue, and three employed Methylene Blue. For the control groups, nine studies used Mechanical Debridement, three used Chlorhexidine, and one used a combination of Mechanical Debridement and Chlorhexidine. Detailed characteristics of the included studies, including participant demographics, definition of peri-implantitis, treatment modalities, and outcome measures, are systematically presented in Table 1.

**Table 1:** Overview of included studies, intervention details, and Peri-Implantitis diagnostic criteria

Study	Subjects (intervention/control)	Sex (male/female)	Mean age (intervention/control)	Definition of Peri-Implantitis	Photosensitizer	Wavelength	Power	Intervention detail		Follow-up	Outcome
								Intervention	Control		
Ahmed et al. (2020)	40 (20/20)	20/0 vs. 20/0	48.9 ± 4.5 vs. 50.7 ± 5.9	PD ≥ 5 mm; ≥ 2 mm bone loss from baseline; presence of BOP and/or suppuration	Phenothiazine chloride, 0.005%	660 nm	150 mW	PDT	MD	6 months	BOP, PD, PI, CBL
Wang et al. (2019)	131 (65/66)	23/42 vs. 25/41	44.1 ± 9.8 vs. 42.6 ± 13.0	PD ≥ 6 mm; ≥ 3 mm or ≥ 25% implant length; clinical signs of bleeding and peri-implant inflammation	Toluidine Blue, 100 mg/ml.	635 nm	750 mW	PDT	MD + CHX	6 months	PD, CAL
Amri et al. (2016)	67 (34/33)	19/15 vs. 17/16	53.6 ± 9.5 vs. 51.4 ± 3.7	PD ≥ 5 mm; ≥ 2 mm alveolar bone loss; BOP present at ≥ 1 peri-implant site	Phenothiazine chloride, 0.005%	660 nm	100 mW	PDT	MD	12 months	BOP, PD, CBL
Al-Askar et al. (2021)	32 (16/16)	13/3 vs. 10/6	65.2 ± 1.3 vs. 62.8 ± 2.5	PD ≥ 4 mm; ≥1.5 mm bone loss from baseline; BOP and clinical signs of edema	Methylene blue, 0.005%	660 nm	180 mW	PDT	MD	3 months	PD, PI, CBL
Alqahtani et al. (2019)	98 (49/49)	49/0 vs. 49/0	52.3 ± 2.2 vs. 54.2 ± 2.2	PD ≥ 5 mm; ≥2 mm bone loss from baseline; persistent BOP, mucosal swelling	Methylene blue, 0.005%	660 nm	150 mW	PDT	MD	6 months	BOP, PD, PI, CBL
Al Rifaiy et al. (2018)	38 (20/18)	20/0 vs. 18/0	33.6 ± 2.8 vs. 35.4 ± 2.1	PD ≥ 5 mm; ≥3 mm radiographic bone loss; BOP and suppuration	Methylene blue, 0.005%	670 nm	150 mW	PDT	CHX	12 weeks	BOP, PD, PI
Deeb et al. (2019)	30 (15/15)	15/0 vs. 15/0	52.6 ± 0.9 vs. 49.2 ± 0.13	PD ≥ 5 mm; ≥2 mm radiographic bone loss; BOP confirmed at consecutive visits	Phenothiazine chloride, 0.005%	660 nm	100 mW	PDT	MD	12 weeks	BOP, PD, PI
Javed et al. (2017)	54 (28/26)	28/0 vs. 26/0	50.6 ± 0.8 vs. 52.2 ± 0.5	PD ≥ 6 mm; ≥3 mm from baseline; BOP and redness of peri-implant tissue	Phenothiazine chloride, 0.005%	660 nm	100 mW	PDT	MD	12 weeks	BOP, PD, PI
Karimi et al. (2016)	20 (10/10)	2/8 vs. 2/8	52.8 ± 7.33 vs. 52.8 ± 7.33	PD ≥ 4 mm; ≥1.5 mm bone loss from previous radiographs; BOP and visible signs of inflammation	Toluidine Blue, 100 mg/ml.	630 nm	2000 mW	PDT	MD	3 months	BOP, PD, CAL
Bassetti et al. (2014)	40 (20/20)	10/10 vs. 10/10	59 ± 12.8 vs. 57 ± 11.5	PD ≥ 5 mm; >2 mm radiographic bone loss; BOP and presence of exudate	Phenothiazine chloride, 0.005%	660 nm	100 mW	PDT	MD	12 months	BOP, PD, CAL
Abduljabbar et al. (2016)	60 (30/30)	30/0 vs. 30/0	50.6 ± 1.4 vs. 51.4 ± 0.6	PD ≥ 5 mm; ≥2 mm alveolar bone loss; persistent BOP	Phenothiazine chloride, 0.005%	660 nm	100 mW	PDT	CHX	6 months	BOP, PD
Li et al. (2013)	30 (15/15)	NA	47 vs. 47	PD ≥ 4 mm; ≥1.5 mm bone loss radiographically; BOP at multiple sites	Toluidine Blue, 100 µg/ml.	635 nm	750 mW	PDT	MD	12 weeks	PD, PI, BI
Wang et al. (2017)	38 (20/18)	9/11 vs. 10/8	43.1 ± 12.2 vs. 43.0 ± 11.7	PD ≥ 5 mm; ≥3 mm from initial follow-up; BOP and mucosal inflammation	Toluidine Blue, 100 µg/ml.	635 nm	750 mW	PDT	CHX	3 months	BOP, PD, PI, BI

PDT: Photodynamic Therapy, MD: Mechanical Debridement, CHX: Chlorhexidine, BOP: Bleeding on Probing, PD: Probing Depth, PI: Plaque Index, CAL: Clinical Attachment Level, CBL: Crestal Bone Loss, BI: Bleeding Index, NA: Not Available or Not Applicable

### Risk of bias

A comprehensive risk of bias assessment was conducted for the thirteen studies included studies. Overall, seven studies were judged to have a low risk of bias, while six studies were assessed as having some concerns. In terms of the randomization process, ten trials (76.9%) were classified as low risk, whereas three trials (23.1%) had some concerns. For deviations from intended interventions, twelve trials (92.3%) were deemed low risk, with only one trial (7.7%) having some concerns. Regarding missing outcome data, ten trials (76.9%) were categorized as low risk, while three trials (23.1%) raised some concerns. All thirteen trials exhibited a low risk of bias in the measurement of outcomes. Concerning the selection of the reported result, twelve trials (92.3%) were classified as low risk, with one trial (7.7%) presenting some concerns. A detailed assessment of the risk of bias across all domains is provided in Table 2.

**Table 2:**
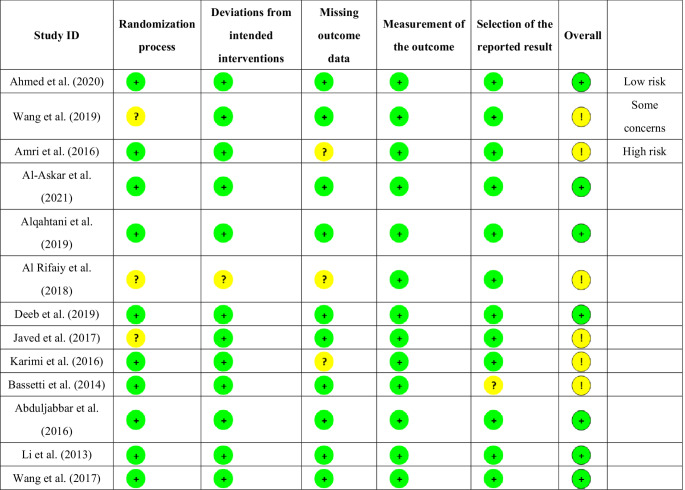
The risk of bias assessment for the individual included studies

### Heterogeneity and sensitivity analysis

Significant heterogeneity was observed across several outcomes, including BOP, PD, PI, and CAL. To address this, sensitivity analyses were conducted (Supplementary File 4), which revealed that in the PD outcome, two studies deviated significantly from the central trend. Similarly, in the PI outcome, one study showed a notable deviation from the central line. For other outcomes, the data points clustered closely around the mean effect size, suggesting the absence of influential outliers. Despite the observed heterogeneity, none of the outlier studies were excluded from the analysis, given their low risk of bias and the relatively small number of included studies.

### Publication bias

Potential publication bias was assessed using funnel plots, with the plots demonstrating varying degrees of symmetry around the vertical axis. Funnel plots for most outcomes (Supplementary Figs. 5.1, 5.4, 5.5, and 5.6) showed near symmetry, suggesting minimal publication bias. However, Supplementary Figs. 5.2 and 5.3 displayed notable asymmetry, indicating potential bias. To further assess this, Egger’s test was performed, and results are discussed in Supplementary File 5.

### The results of meta-analysis

#### BOP

**Fig. 2:**
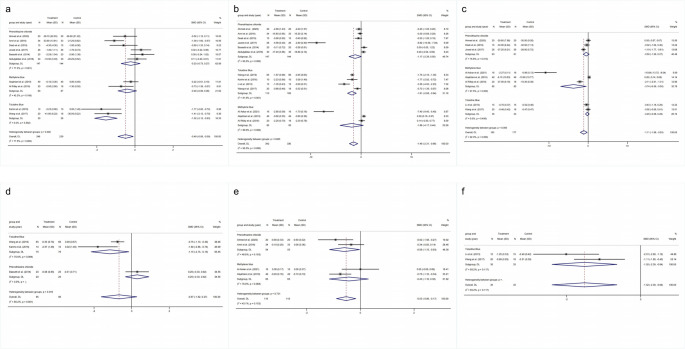
Forest plot of the efficacy for **a**: BOP, **b**: PD, **c**: PI, **d**: CAL, **e**: CBL, **f**: BI

A total of ten studies encompassing 485 patients were included in the meta-analysis to assess the impact of PDT on BOP in peri-implantitis patients. Among them, four studies used Toluidine Blue, four used Phenothiazine chloride, and two used Methylene Blue. The meta-analysis demonstrated a significant reduction in BOP in the PDT group compared to controls (SMD = −0.49, 95% CI: −0.89, −0.09) (Fig. 2a), suggesting that PDT improves peri-implant health. Subgroup analysis revealed variability in efficacy across different photosensitizers. Specifically, PDT using Toluidine Blue significantly improved BOP (SMD = −1.52, 95% CI: −2.12, −0.93), while no significant changes were observed in groups treated with Phenothiazine chloride (SMD = −0.23, 95% CI: −0.73, 0.27) or Methylene Blue (SMD = −0.40, 95% CI: −0.89, 0.08).

#### PD

Thirteen studies involving 678 patients were analyzed to evaluate the effects of PDT on PD. Specifically, four studies used Toluidine Blue, six studies used Phenothiazine chloride, and three used Methylene Blue. The meta-analysis revealed a significant reduction in PD for the PDT group compared to the control group (SMD = −1.49, 95% CI: −2.31, −0.66) (Fig. 2b). Subgroup analysis showed that the efficacy of PDT varied with different photosensitizers. Notably, the Toluidine Blue subgroup demonstrated a significant improvement in PD (SMD = −1.81, 95% CI: −2.68, −0.94). In contrast, the subgroups using Phenothiazine chloride (SMD = −1.17, 95% CI: −2.39, 0.05) and Methylene blue (SMD = −1.86, 95% CI: −4.17, 0.44) did not show statistically significant differences.

#### PI

The meta-analysis included 8 studies with a total of 360 patients to assess the impact of PDT on PI. This included two studies using Toluidine Blue, four using Phenothiazine chloride, and two using Methylene Blue. The overall effect size indicated that PDT significantly reduced PI compared to the control group (SMD = −1.11, 95% CI: −1.98, −0.25) (Fig. 2c). Subgroup analysis revealed that Methylene blue was particularly effective, showing a substantial reduction in PI (SMD = −3.74, 95% CI: −6.99, −0.50). Conversely, the Phenothiazine chloride (SMD = −0.50, 95% CI: −1.26, 0.27) and Toluidine Blue (SMD = −0.20, 95% CI: −0.68, 0.28) subgroups did not exhibit significant differences.

#### CAL

Three studies comprising 191 patients were included in the analysis of PDT’s effects on CAL. Among them, one study used Toluidine Blue and two studies used Phenothiazine chloride. The overall meta-analysis did not find a statistically significant difference between the PDT and control groups (SMD = −0.67, 95% CI: −1.62, 0.27) (Fig. 2d). However, subgroup analysis showed that PDT using Toluidine Blue significantly improved CAL (SMD = −1.15, 95% CI: −2.15, −0.15). In contrast, the Phenothiazine chloride subgroup did not show a statistically significant difference (SMD = 0.29, 95% CI: −0.33, 0.92).

#### CBL

The meta-analysis included four studies with a total of 237 patients to evaluate the effects of PDT on CBL. Of these, three studies used Phenothiazine chloride and one study used Methylene Blue. The pooled results indicated a significant reduction in CBL in the PDT group compared to the control group (SMD = −0.53, 95% CI: −0.89, −0.17) (Fig. 2e). Subgroup analysis further highlighted that the Phenothiazine chloride subgroup experienced a significant improvement in CBL (SMD = −0.59, 95% CI: −1.15, −0.03). However, the Methylene blue subgroup did not demonstrate a significant difference (SMD = −0.43, 95% CI: −1.16, 0.30).

#### BI

Two studies involving 68 patients were analyzed to assess the impact of PDT on BI, both utilizing Toluidine Blue as the photosensitizer. The meta-analysis indicated a significant reduction in BI in the PDT group compared to the control group (SMD = −1.52, 95% CI: −2.39, −0.64) (Fig. 2f).

## Discussion

The present meta-analysis, which synthesized data from thirteen RCTs, provides a comprehensive evaluation of the efficacy of PDT in the treatment of peri-implantitis. The primary findings of this study indicate that PDT is significantly effective in improving clinical outcomes for patients with peri-implantitis. Our findings indicate that PDT significantly improves key clinical outcomes, including BOP, PD, PI, CBL, and BI—all critical indicators of peri-implant health and treatment success. Furthermore, this study highlights the differential efficacy of various photosensitizers used in PDT. Among the various photosensitizers, Toluidine Blue demonstrated the most pronounced efficacy, significantly improving BOP, PD, CAL, and BI. Meanwhile, Methylene Blue exhibited a notable effect in reducing PI, and Phenothiazine chloride was effective in mitigating CBL. These findings underscore the potential of PDT as a valuable adjunctive treatment in managing peri-implantitis, offering a less invasive alternative to conventional therapies. This study’s significance lies in its ability to delineate the specific benefits of different photosensitizers, thereby providing evidence-based guidance for optimizing PDT protocols in clinical practice.

In particular, our meta-analysis reveals that while PDT demonstrates significant efficacy in improving most peri-implantitis-related clinical outcomes, it did not show a statistically significant effect on CAL. However, PDT’s positive impact on other critical indicators, such as BOP, PD, PI, CBL, and BI, underscores its overall effectiveness. These findings are consistent with the broader literature on the subject [30]. For instance, a systematic review by Alqahtani et al. (2019) concluded that PDT significantly improved clinical parameters such as probing depth and bleeding on probing in peri-implantitis patients compared to mechanical debridement alone [23]. Similarly, Zhao et al. (2020) emphasized the short-term benefits of PDT as an adjunct therapy, although they highlighted a lack of standardized protocols across studies. Our findings align with these observations and further extend the literature by evaluating the comparative effectiveness of different photosensitizers in PDT [31]. For example, Renvert et al. reported that PDT significantly reduces peri-implant inflammation and microbial load, aligning with our observation of substantial improvements in BOP and PD [32]. Similarly, the study by Kotsakis et al. demonstrated that PDT has a notable advantage in reducing probing depth and plaque index, which corroborates our results [33]. These comparative studies further validate our conclusion that PDT is an effective adjunctive therapy for managing peri-implantitis, offering significant benefits across multiple clinical outcomes, except for CAL.

The advantages of PDT in treating peri-implantitis may be related to several proposed mechanisms. Firstly, PDT is believed to exert antimicrobial effects by generating reactive oxygen species (ROS), which may contribute to biofilm disruption and reduced inflammation [34, 35]. Secondly, experimental studies have suggested that PDT may be associated with cellular responses conducive to tissue repair, such as fibroblast and osteoblast activity [36], although such effects have not been consistently confirmed in human RCTs with histological endpoints. Thirdly, PDT might help improve local circulation, which could support the resolution of inflammation and tissue recovery [37] While these biological mechanisms are plausible, the current clinical evidence does not permit definitive conclusions regarding tissue regeneration, and further mechanistic studies are warranted.

Despite the overall positive findings, the heterogeneity observed in the results necessitated a further subgroup analysis based on the different photosensitizers used in PDT. Specifically, Toluidine Blue demonstrated significant improvements in BOP, PD, CAL, and BI, with support from four RCTs; Methylene Blue showed a notable effect in reducing PI across three RCTs; and Phenothiazine chloride was associated with improved CBL in six RCTs. However, caution is warranted in interpreting these subgroup findings, especially for Methylene Blue and Toluidine Blue, as the number of supporting studies was limited, potentially reducing statistical power. Comparatively, Toluidine Blue appears to provide the broadest clinical benefit, especially in both soft tissue and inflammatory indices. Methylene Blue’s strength lies primarily in plaque reduction (PI), while Phenothiazine chloride may be more beneficial in preserving crestal bone structure (CBL). These relative advantages suggest that the selection of photosensitizer should consider the specific clinical objectives of PDT. These findings corroborate previous studies, which have also highlighted the varying effectiveness of different photosensitizers in PDT [37, 38]. The differential efficacy of these agents can be attributed to several factors. Toluidine Blue, for instance, has a strong affinity for bacterial cell walls, enhancing its ability to produce ROS and effectively reduce microbial load and inflammation [39]. Methylene Blue’s efficacy in reducing PI may be due to its optimal absorption properties and its ability to penetrate biofilms, making it particularly effective against plaque-associated bacteria [40]. Phenothiazine chloride’s impact on CBL could be linked to its dual role in reducing bacterial load and promoting osteoblastic activity, thereby contributing to bone preservation [41]. These insights emphasize that the choice of photosensitizer is crucial in optimizing PDT outcomes, and tailoring the photosensitizer to the specific clinical scenario may enhance the overall effectiveness of peri-implantitis treatment.

This study presents several strengths that enhance the robustness and reliability of its findings. Firstly, the use of a rigorous network meta-analysis approach allows for a comprehensive comparison of the effectiveness of different photosensitizers in PDT, providing a more nuanced understanding of their relative efficacy. Secondly, the inclusion of high-quality RCTs ensures that the evidence synthesized is based on methodologically sound studies, thereby increasing the credibility of the results. However, there are also some limitations that should be acknowledged. Firstly, the heterogeneity in intervention protocols across the included studies, such as variations in the type of laser technology and differences in the duration and frequency of PDT sessions, could have influenced the outcomes, potentially leading to variability in the results. Secondly, the dosage and application protocols of photosensitizers were not standardized across studies. Variations in concentration, exposure time, and light delivery parameters may have introduced further methodological heterogeneity, potentially influencing treatment efficacy and limiting direct comparability. Thirdly, the relatively short follow-up periods in some trials may not fully capture the long-term effects of PDT on peri-implant health, limiting the ability to assess the sustainability of the observed benefits. Lastly, the study population was restricted to patients with peri-implantitis, which may constrain the generalizability of the findings to broader populations, such as those with less severe peri-implant conditions or those in different demographic groups. In addition, several disadvantages and potential adverse effects of PDT should be noted. Some included studies reported mild and transient side effects, such as local discomfort, soft tissue irritation, or burning sensations following light activation. The risk of mucosal or dental staining—particularly with photosensitizers like Toluidine Blue and Methylene Blue—may affect patient acceptance [42]. Furthermore, the efficacy of PDT is highly operator-dependent, requiring precise light delivery and uniform photosensitizer application; insufficient technical performance could reduce clinical effectiveness. The requirement for specialized devices and trained personnel may also limit its accessibility and routine integration into general dental practice. Given these limitations, future research should focus on standardizing PDT treatment protocols, including optimal laser parameters and photosensitizer selection. Additionally, long-term studies with extended follow-up periods are necessary to assess the sustained effects of PDT on peri-implant health. Expanding research to include diverse patient populations and varying stages of peri-implant disease would also help determine the broader applicability of PDT in clinical practice.

## Conclusion

This meta-analysis demonstrates that PDT is an effective treatment for peri-implantitis, significantly improving key clinical outcomes such as BOP, PD, PI, CBL, and BI. The effectiveness of PDT varies with different photosensitizers, with Toluidine Blue showing the most comprehensive benefits. Despite some heterogeneity and limitations in study design, PDT’s ability to reduce inflammation and promote tissue healing positions it as a valuable adjunct in peri-implant therapy. Future research should focus on standardizing treatment protocols and assessing the long-term efficacy of PDT across diverse patient populations to enhance its clinical application.

## Supplementary Information

Below is the link to the electronic supplementary material.

Supplementary Material 1

## Data Availability

All data generated or analysed during this study are included in this published article and its supplementary information files.
