# Supplementary material for: Efficacy of photodynamic therapy with various photosensitizers for peri-implantitis treatment: a systematic review and meta-analysis of randomized clinical trials
Source: Lasers Med Sci. 2025 Sep 16;40(1):359. doi: 10.1007/s10103-025-04612-7 (PMC12441095; doi:10.1007/s10103-025-04612-7)
Supplement: Supplementary file 1 — Supplementary Material 1 [file 10103_2025_4612_MOESM1_ESM.docx]

**Supplementary**

Table of Contents:

**[Supplementary 1: Search Strategy 2](#_Toc159076731)**

**[Supplementary 2:](#_Toc159076731)** [Characteristics of studies and subjects included in the review](#_Toc159076731) **[6](#_Toc159076731)**

[Supplementary 3: Sensitivity analysis 9](#_Toc159076747)

[Supplementary 4: Publication bias](#_Toc159076752) 11

# Supplementary 1: Search Strategy

***Search Strategy (PubMed):***

| #8 | Search: ((((((((((Peri-Implantitis[MeSH Terms]) OR (Peri-Implantitis[Title/Abstract])) OR (Periimplantitis[Title/Abstract])) OR ("Peri-implant Disease"[Title/Abstract])) OR ("Peri-implant Infection"[Title/Abstract])) OR (Implantitis[Title/Abstract])) OR ("Peri-implant Bone Loss"[Title/Abstract])) OR ("Peri-implant Mucositis"[Title/Abstract])) OR ("Peri-implant Inflammatory Disease"[Title/Abstract])) AND (((Photochemotherapy[MeSH Terms])) OR ((((((("Phototherapy"[Title/Abstract])) OR ("Photopharmacology"[Title/Abstract])) OR ("Photodynamic Therapy"[Title/Abstract])) OR ("Light Therapy"[Title/Abstract])) OR ("Actinotherapy"[Title/Abstract])) OR ("Photoirradiation Therapy"[Title/Abstract]) OR ("Photoactive Therapy"[Title/Abstract])))) AND ((randomized controlled trial[pt] OR controlled clinical trial[pt] OR randomized[tiab] OR placebo[tiab] OR drug therapy[sh] OR randomly[tiab] OR trial[tiab] OR groups[tiab]) NOT (animals[mh] NOT humans[mh])) |
| --- | --- |
| #7 | Search: (randomized controlled trial[pt] OR controlled clinical trial[pt] OR randomized[tiab] OR placebo[tiab] OR drug therapy[sh] OR randomly[tiab] OR trial[tiab] OR groups[tiab]) NOT (animals[mh] NOT humans[mh]) |
| #6 | Search: ((Photochemotherapy[MeSH Terms])) OR ((((((("Phototherapy"[Title/Abstract])) OR ("Photopharmacology"[Title/Abstract])) OR ("Photodynamic Therapy"[Title/Abstract])) OR ("Light Therapy"[Title/Abstract])) OR ("Actinotherapy"[Title/Abstract])) OR ("Photoirradiation Therapy"[Title/Abstract]) OR ("Photoactive Therapy"[Title/Abstract])) |
| #5 | Search: (((((("Phototherapy"[Title/Abstract])) OR ("Photopharmacology"[Title/Abstract])) OR ("Photodynamic Therapy"[Title/Abstract])) OR ("Light Therapy"[Title/Abstract])) OR ("Actinotherapy"[Title/Abstract])) OR ("Photoirradiation Therapy"[Title/Abstract]) OR ("Photoactive Therapy"[Title/Abstract]) |
| #4 | Search: (Photochemotherapy[MeSH Terms]) |
| #3 | Search: ((((((((Peri-Implantitis[MeSH Terms]) OR (Peri-Implantitis[Title/Abstract])) OR (Periimplantitis[Title/Abstract])) OR ("Peri-implant Disease"[Title/Abstract])) OR ("Peri-implant Infection"[Title/Abstract])) OR (Implantitis[Title/Abstract])) OR ("Peri-implant Bone Loss"[Title/Abstract])) OR ("Peri-implant Mucositis"[Title/Abstract])) OR ("Peri-implant Inflammatory Disease"[Title/Abstract]) |
| #2 | Search: (((((((Peri-Implantitis[Title/Abstract])) OR (Periimplantitis[Title/Abstract])) OR ("Peri-implant Disease"[Title/Abstract])) OR ("Peri-implant Infection"[Title/Abstract])) OR (Implantitis[Title/Abstract])) OR ("Peri-implant Bone Loss"[Title/Abstract])) OR ("Peri-implant Mucositis"[Title/Abstract])) OR ("Peri-implant Inflammatory Disease"[Title/Abstract]) |
| #1 | Search: (Peri-Implantitis[MeSH Terms]) |

***Search Strategy (Embase):***

#1 'peri-implantitis'/exp OR 'peri-implantitis':ti,ab OR 'periimplantitis':ti,ab OR 'peri-implant disease':ti,ab OR 'peri-implant infection':ti,ab OR 'implantitis':ti,ab OR 'peri-implant bone loss':ti,ab OR 'peri-implant mucositis':ti,ab OR 'peri-implant inflammatory disease':ti,ab

#2 'photochemotherapy'/exp OR 'photochemotherapy':ti,ab OR 'phototherapy':ti,ab OR 'photopharmacology':ti,ab OR 'photodynamic therapy':ti,ab OR 'light therapy':ti,ab OR 'actinotherapy':ti,ab OR 'photoirradiation therapy':ti,ab OR 'photoactive therapy':ti,ab

#3 'randomized controlled trial'/exp OR 'controlled clinical trial'/exp OR random*:ti,ab OR placebo:ti,ab OR 'drug therapy'/exp OR trial:ti,ab OR groups:ti,ab

#4 NOT ([animals]/lim NOT [humans]/lim)

#5 #1 AND #2 AND #3 AND #4

***Search Strategy (Web of Science):***

#1 TS=("Peri-Implantitis" OR "Periimplantitis" OR "Peri-implant Disease" OR "Peri-implant Infection" OR "Implantitis" OR "Peri-implant Bone Loss" OR "Peri-implant Mucositis" OR "Peri-implant Inflammatory Disease")

#2 TS=("Photochemotherapy" OR "Phototherapy" OR "Photopharmacology" OR "Photodynamic Therapy" OR "Light Therapy" OR "Actinotherapy" OR "Photoirradiation Therapy" OR "Photoactive Therapy")

#3 TS=(randomized OR randomly OR placebo OR "controlled clinical trial" OR "randomized controlled trial" OR trial OR groups)

#4 #1 AND #2 AND #3

#5 Refined by: DOCUMENT TYPES = (ARTICLE OR REVIEW) AND LANGUAGES = (ENGLISH)

# Supplementary 2: Characteristics of studies and subjects included in the review

| **Study** | **Subjects  (intervention/ control)** | **Sex (male/female)** | **Mean age  (intervention/ control)** | **Photosensitizer** | **Wavelength** | **Power** | **Intervention detail** | | **Follow-up** | **Outcome** |
| --- | --- | --- | --- | --- | --- | --- | --- | --- | --- | --- |
|  |  |  |  |  |  |  | **Intervention** | **Control** |  |  |
| Ahmed et al. (2020) | 40 (20/20) | 20/0 vs. 20/0 | 48.9±4.5 vs. 50.7±5.9 | Phenothiazine chloride, 0.005% | 660 nm | 150 mW | PDT | MD | 6 months | BOP, PD, PI, CBL |
| Wang et al. (2019) | 131 (65/66) | 23/42 vs. 25/41 | 44.1±9.8 vs. 42.6±13.0 | Toluidine Blue, 100 mg/ml. | 635 nm | 750 mW | PDT | MD+CHX | 6 months | PD, CAL |
| Amri et al. (2016) | 67 (34/33) | 19/15 vs. 17/16 | 53.6±9.5 vs. 51.4±3.7 | Phenothiazine chloride, 0.005% | 660 nm | 100 mW | PDT | MD | 12 months | BOP, PD, CBL |
| Al-Askar et al. (2021) | 32 (16/16) | 13/3 vs. 10/6 | 65.2±1.3 vs. 62.8±2.5 | Methylene blue, 0.005% | 660 nm | 180 mW | PDT | MD | 3 months | PD, PI, CBL |
| Alqahtani et al. (2019) | 98 (49/49) | 49/0 vs. 49/0 | 52.3±2.2 vs. 54.2±2.2 | Methylene blue, 0.005% | 660 nm | 150 mW | PDT | MD | 6 months | BOP, PD, PI, CBL |
| Al Rifaiy et al. (2018) | 38 (20/18) | 20/0 vs. 18/0 | 33.6±2.8 vs. 35.4±2.1 | Methylene blue, 0.005% | 670 nm | 150 mW | PDT | CHX | 12 weeks | BOP, PD, PI |
| Deeb et al. (2019) | 30 (15/15) | 15/0 vs. 15/0 | 52.6±0.9 vs. 49.2±0.13 | Phenothiazine chloride, 0.005% | 660 nm | 100 mW | PDT | MD | 12 weeks | BOP, PD, PI |
| Javed et al. (2017) | 54 (28/26) | 28/0 vs. 26/0 | 50.6±0.8 vs. 52.2±0.5 | Phenothiazine chloride, 0.005% | 660 nm | 100 mW | PDT | MD | 12 weeks | BOP, PD, PI |
| Karimi et al. (2016) | 20 (10/10) | 2/8 vs. 2/8 | 52.8±7.33 vs. 52.8±7.33 | Toluidine Blue, 100 mg/ml. | 630 nm | 2000 mW | PDT | MD | 3 months | BOP, PD, CAL |
| Bassetti et al. (2014) | 40 (20/20) | 10/10 vs. 10/10 | 59±12.8 vs. 57±11.5 | Phenothiazine chloride, 0.005% | 660 nm | 100 mW | PDT | MD | 12 months | BOP, PD, CAL |
| Abduljabbar et al. (2016) | 60 (30/30) | 30/0 vs. 30/0 | 50.6±1.4 vs. 51.4±0.6 | Phenothiazine chloride, 0.005% | 660 nm | 100 mW | PDT | CHX | 6 months | BOP, PD |
| Li et al. (2013) | 30 (15/15) | NA | 47 vs. 47 | Toluidine Blue, 100 µg/ml. | 635 nm | 750 mW | PDT | MD | 12 weeks | PD, PI, BI |
| Wang et al. (2017) | 38 (20/18) | 9/11 vs. 10/8 | 43.1±12.2 vs. 43.0±11.7 | Toluidine Blue, 100 µg/ml. | 635 nm | 750 mW | PDT | CHX | 3 months | BOP, PD, PI, BI |

Note: PDT, Photodynamic Therapy; MD, Mechanical Debridement; CHX, Chlorhexidine; BOP, Bleeding on Probing; PD, Probing Depth; PI, Plaque Index; CAL, Clinical Attachment Level; CBL, Crestal Bone Loss; BI, Bleeding Index, NA, Not Applicable.

**List of included studies:**

1. Ahmed P, Bukhari IA, Albaijan R, Sheikh SA, Vohra F. The effectiveness of photodynamic and antibiotic gel therapy as an adjunct to mechanical debridement in the treatment of peri-implantitis among diabetic patients. Photodiagnosis Photodyn Ther. 2020 Dec;32:102077. doi: 10.1016/j.pdpdt.2020.102077. Epub 2020 Nov 4. PMID: 33157330.
2. Wang H, Li W, Zhang D, Li W, Wang Z. Adjunctive photodynamic therapy improves the outcomes of peri-implantitis: a randomized controlled trial. Aust Dent J. 2019 Sep;64(3):256-262. doi: 10.1111/adj.12705. Epub 2019 Jun 21. PMID: 31152567.
3. Al Amri MD, Kellesarian SV, Ahmed A, Al-Kheraif AA, Romanos GE, Javed F. Efficacy of periimplant mechanical debridement with and without adjunct antimicrobial photodynamic therapy in patients with type 2 diabetes mellitus. Photodiagnosis Photodyn Ther. 2016 Jun;14:166-9. doi: 10.1016/j.pdpdt.2016.04.015. Epub 2016 Apr 26. PMID: 27129871.
4. Al-Askar MH, Abdullatif FA, Alshihri AA, Ahmed A, Divakar DD, Almoharib H, Alzoman H. Comparison of photobiomodulation and photodynamic therapy as adjuncts to mechanical debridement for the treatment of peri-implantitis. Technol Health Care. 2022;30(2):389-398. doi: 10.3233/THC-213062. PMID: 34250918.
5. Alqahtani F, Alqhtani N, Alkhtani F, Divakar DD, Al-Kheraif AA, Javed F. Efficacy of mechanical debridement with and without adjunct antimicrobial photodynamic therapy in the treatment of peri-implantitis among moderate cigarette-smokers and waterpipe-users. Photodiagnosis Photodyn Ther. 2019 Dec;28:153-158. doi: 10.1016/j.pdpdt.2019.09.003. Epub 2019 Sep 13. PMID: 31525451.
6. Al Rifaiy MQ, Qutub OA, Alasqah MN, Al-Sowygh ZH, Mokeem SA, Alrahlah A. Effectiveness of adjunctive antimicrobial photodynamic therapy in reducing peri-implant inflammatory response in individuals vaping electronic cigarettes: A randomized controlled clinical trial. Photodiagnosis Photodyn Ther. 2018 Jun;22:132-136. doi: 10.1016/j.pdpdt.2018.03.002. Epub 2018 Mar 15. PMID: 29550362.
7. Deeb MA, Alsahhaf A, Mubaraki SA, Alhamoudi N, Al-Aali KA, Abduljabbar T. Clinical and microbiological outcomes of photodynamic and systemic antimicrobial therapy in smokers with peri-implant inflammation. Photodiagnosis Photodyn Ther. 2020 Mar;29:101587. doi: 10.1016/j.pdpdt.2019.101587. Epub 2019 Nov 2. PMID: 31689510.
8. Javed F, BinShabaib MS, Alharthi SS, Qadri T. Role of mechanical curettage with and without adjunct antimicrobial photodynamic therapy in the treatment of peri-implant mucositis in cigarette smokers: A randomized controlled clinical trial. Photodiagnosis Photodyn Ther. 2017 Jun;18:331-334. doi: 10.1016/j.pdpdt.2017.04.015. Epub 2017 Apr 27. PMID: 28457847.
9. Karimi MR, Hasani A, Khosroshahian S. Efficacy of Antimicrobial Photodynamic Therapy as an Adjunctive to Mechanical Debridement in the Treatment of Peri-implant Diseases: A Randomized Controlled Clinical Trial. J Lasers Med Sci. 2016 Summer;7(3):139-145. doi: 10.15171/jlms.2016.24. Epub 2016 Jul 18. PMID: 28144432; PMCID: PMC5262478.
10. Bassetti M, Schär D, Wicki B, Eick S, Ramseier CA, Arweiler NB, Sculean A, Salvi GE. Anti-infective therapy of peri-implantitis with adjunctive local drug delivery or photodynamic therapy: 12-month outcomes of a randomized controlled clinical trial. Clin Oral Implants Res. 2014 Mar;25(3):279-287. doi: 10.1111/clr.12155. Epub 2013 Apr 8. PMID: 23560645.
11. Abduljabbar T. Effect of mechanical debridement with and without adjunct antimicrobial photodynamic therapy in the treatment of peri-implant diseases in prediabetic patients. Photodiagnosis Photodyn Ther. 2017 Mar;17:9-12. doi: 10.1016/j.pdpdt.2016.10.011. Epub 2016 Oct 29. PMID: 27989865.
12. Li Y, Fa Y, Yang Y, Cai X, Li G. Treatment of Peri-implantitis with Photodynamic Therapy. J Pract Stomatol. 2013 Nov;29(6):848-850. doi: 10.3969/j.issn.1001-3733.2013.06.022.
13. Wang Y, Ma Y, Liu X, Guo L. Short-term Clinical Effects on the Treatment of Peri-implantitis with Photodynamic Therapy. Henan Medical Research. 2017 Aug;26(16):2890-2892. doi: 10.3969/j.issn.1004-437X.2017.16.003.

# Supplementary 3: Sensitivity analysis


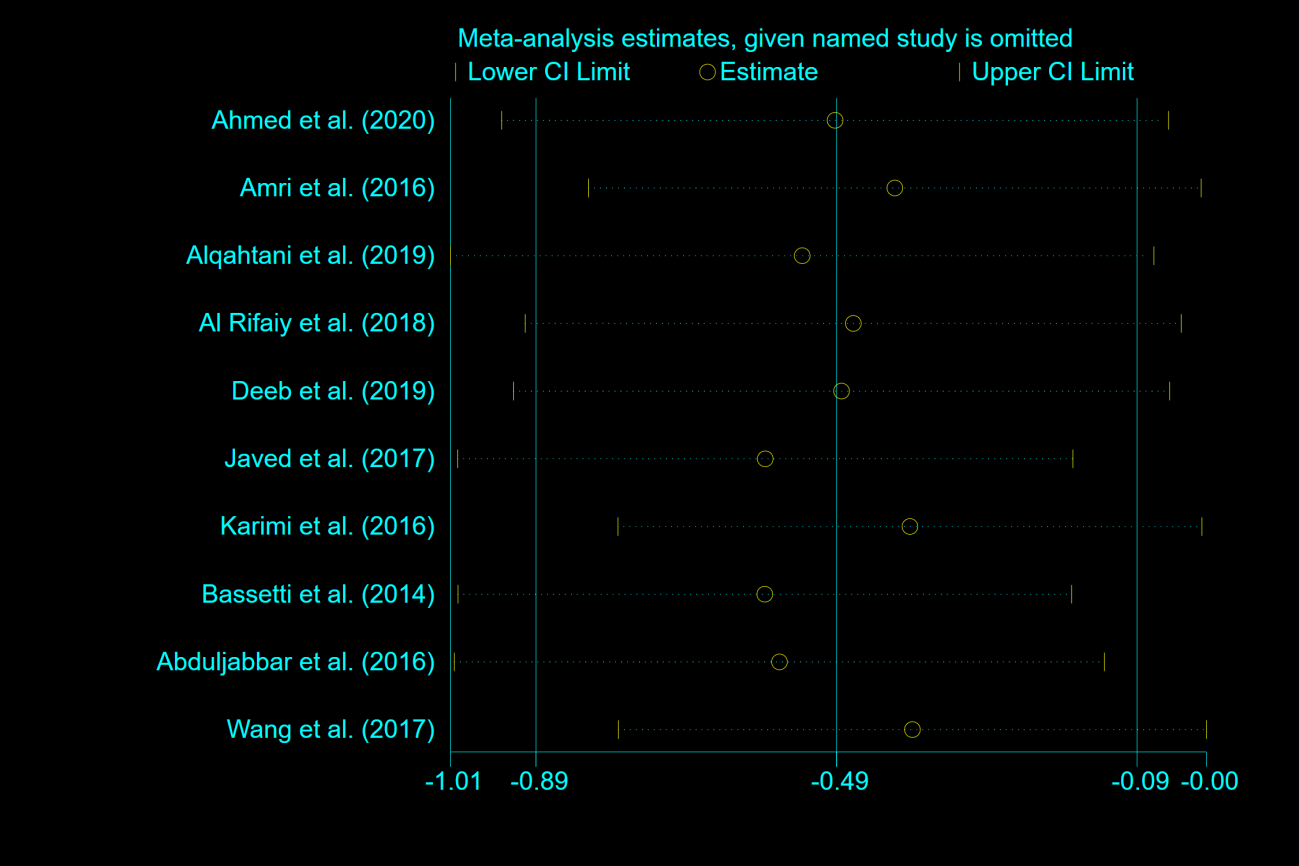


Figure 4.1 The sensitivity analysis of BOP.


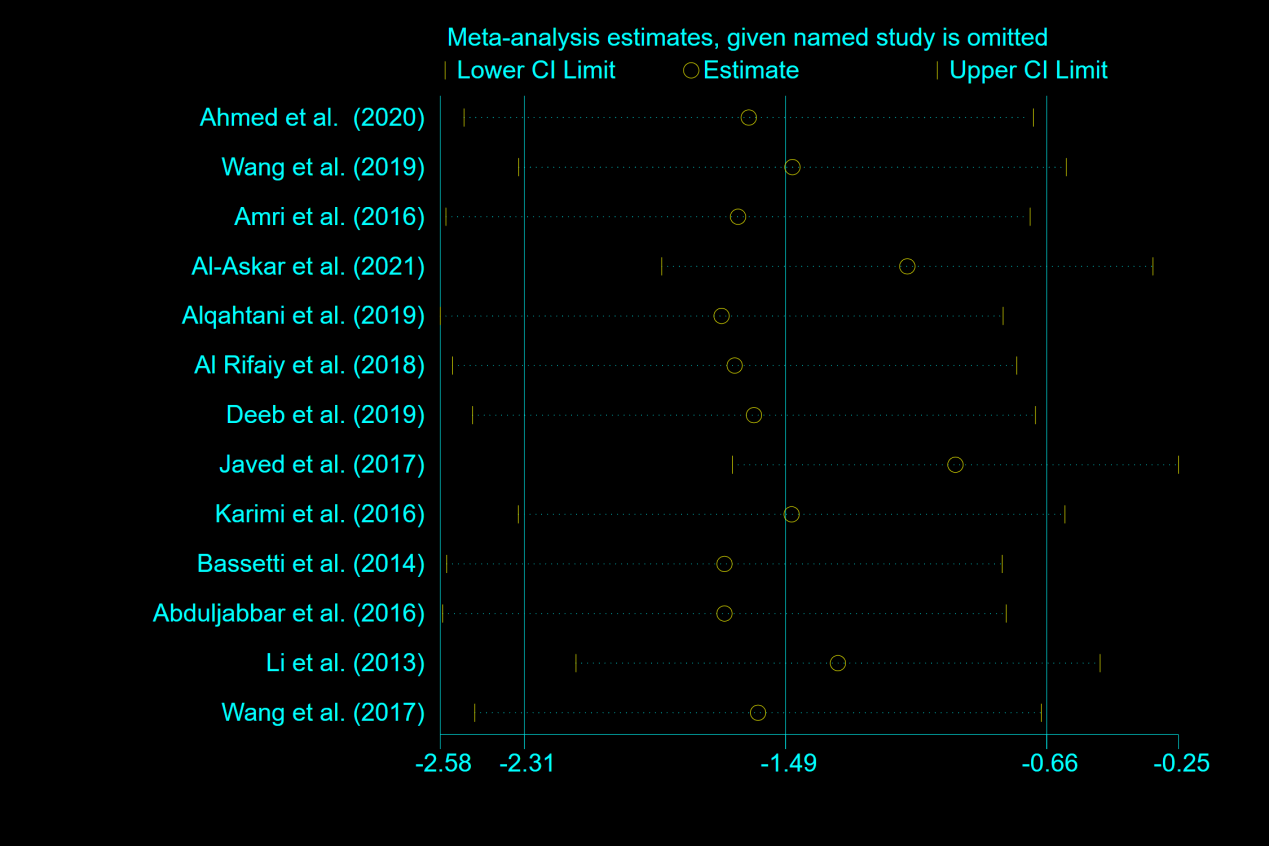


Figure 4.2 The sensitivity analysis of PD.


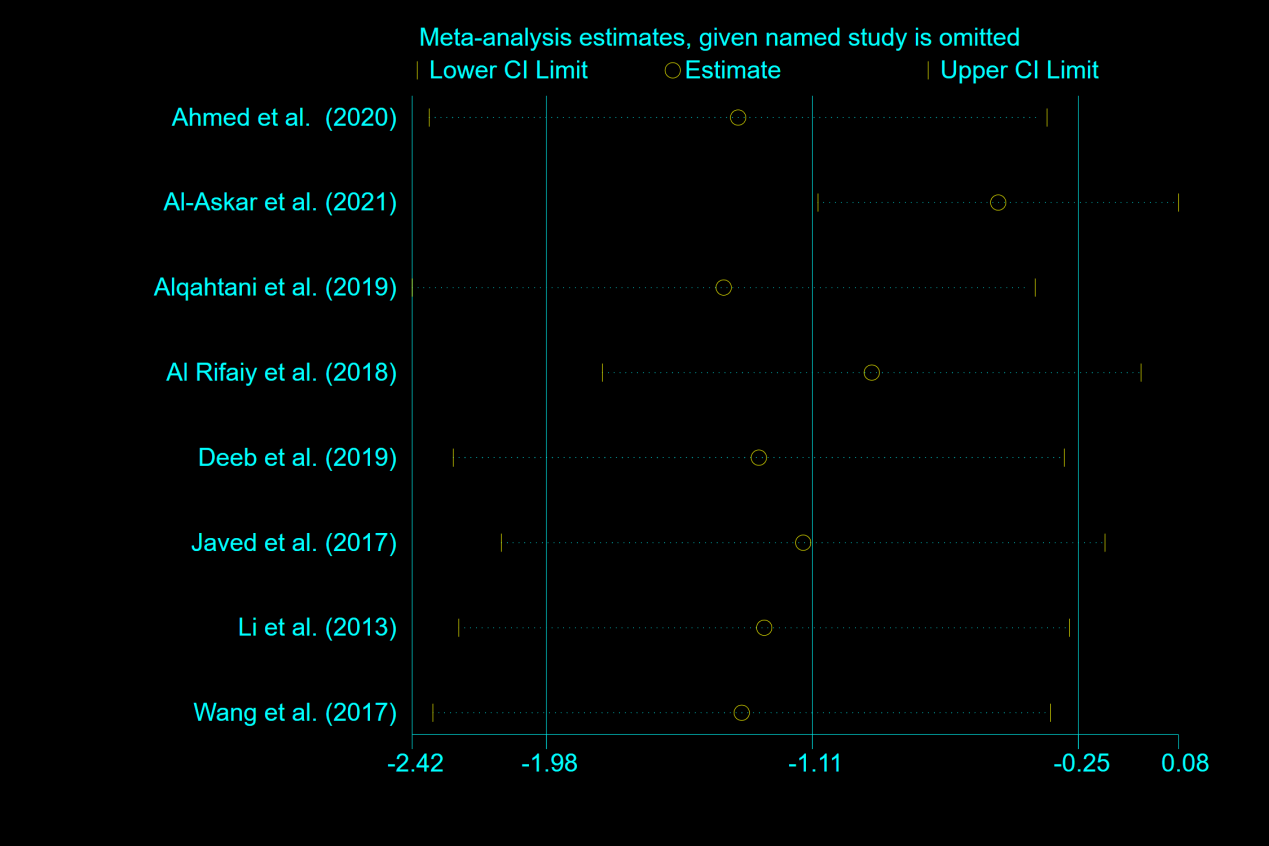


Figure 4.3 The sensitivity analysis of PI.


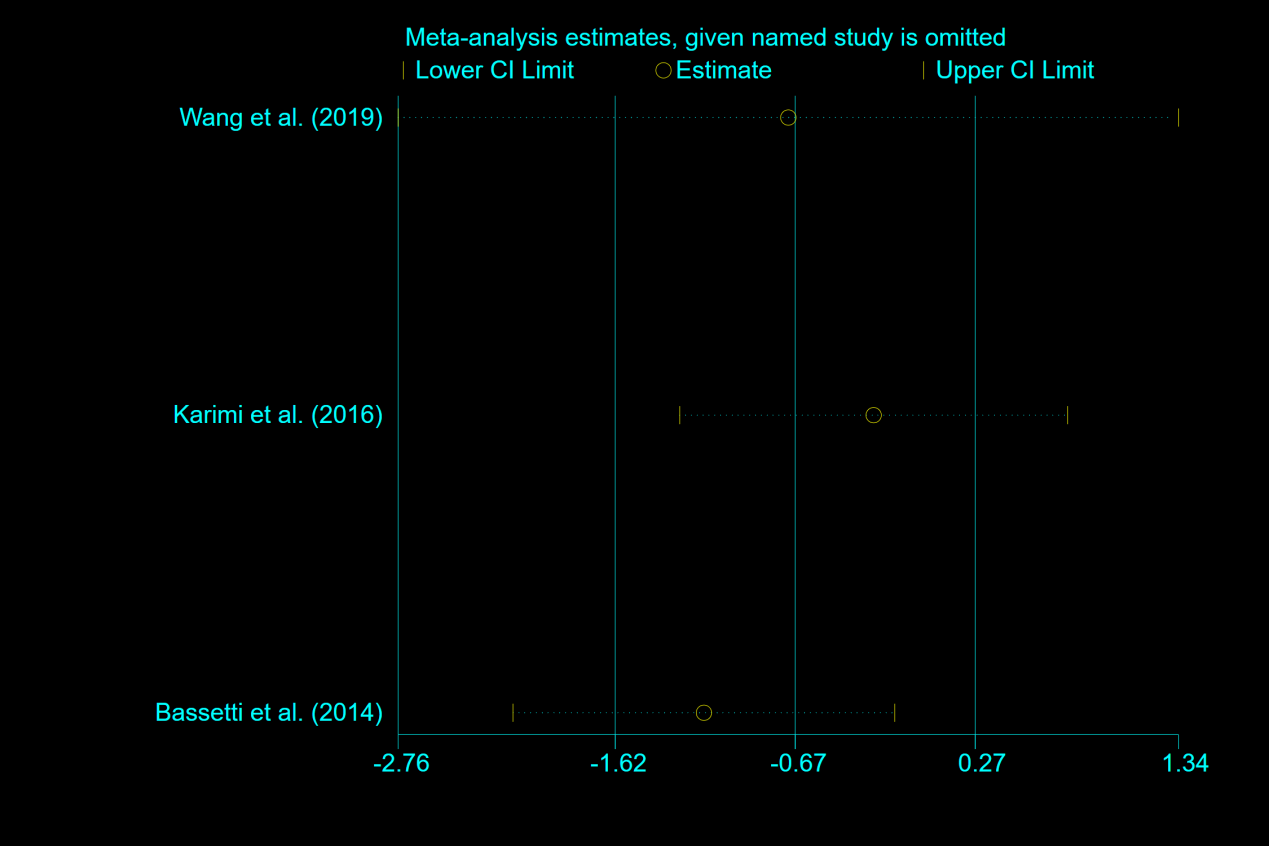


Figure 4.4 The sensitivity analysis of CAL.

# Supplementary 4: Publication bias


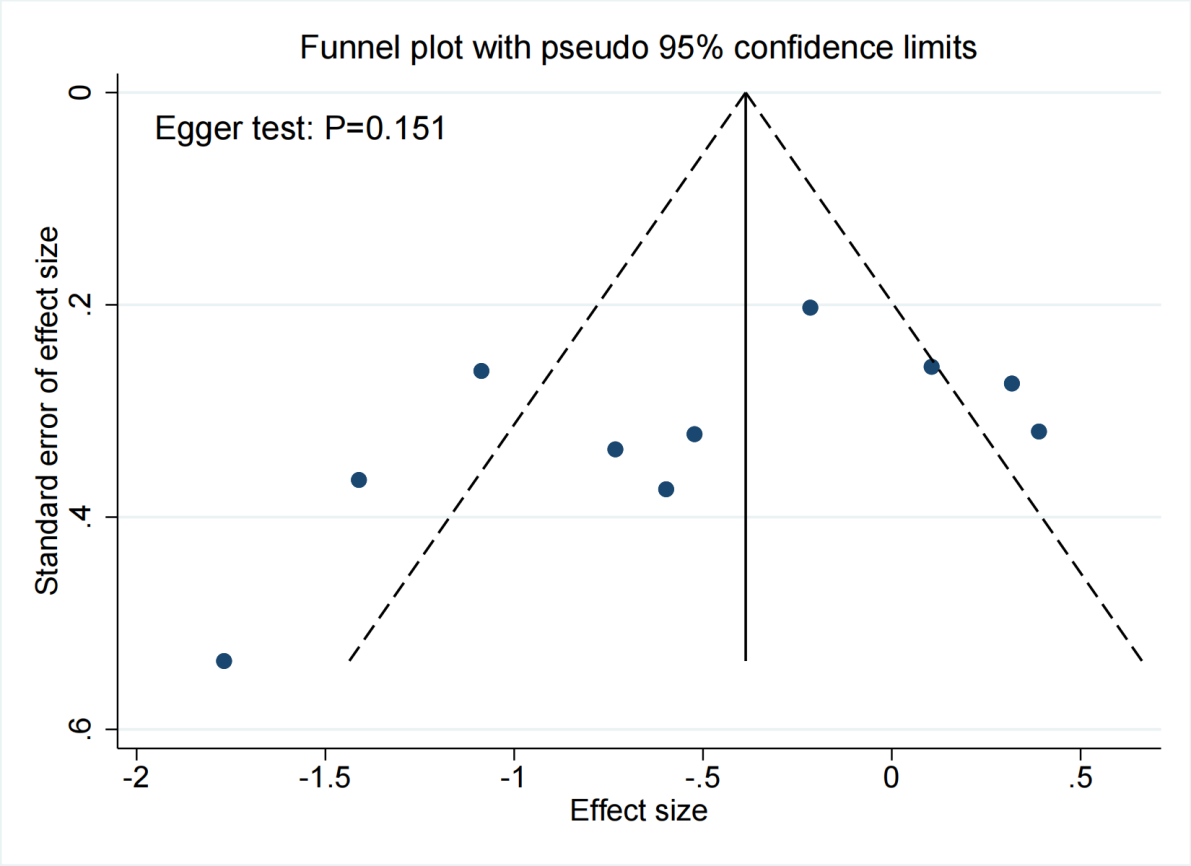


Figure 5.1 The funnel plot of BOP. The result of Egger test showed the p=0.151.


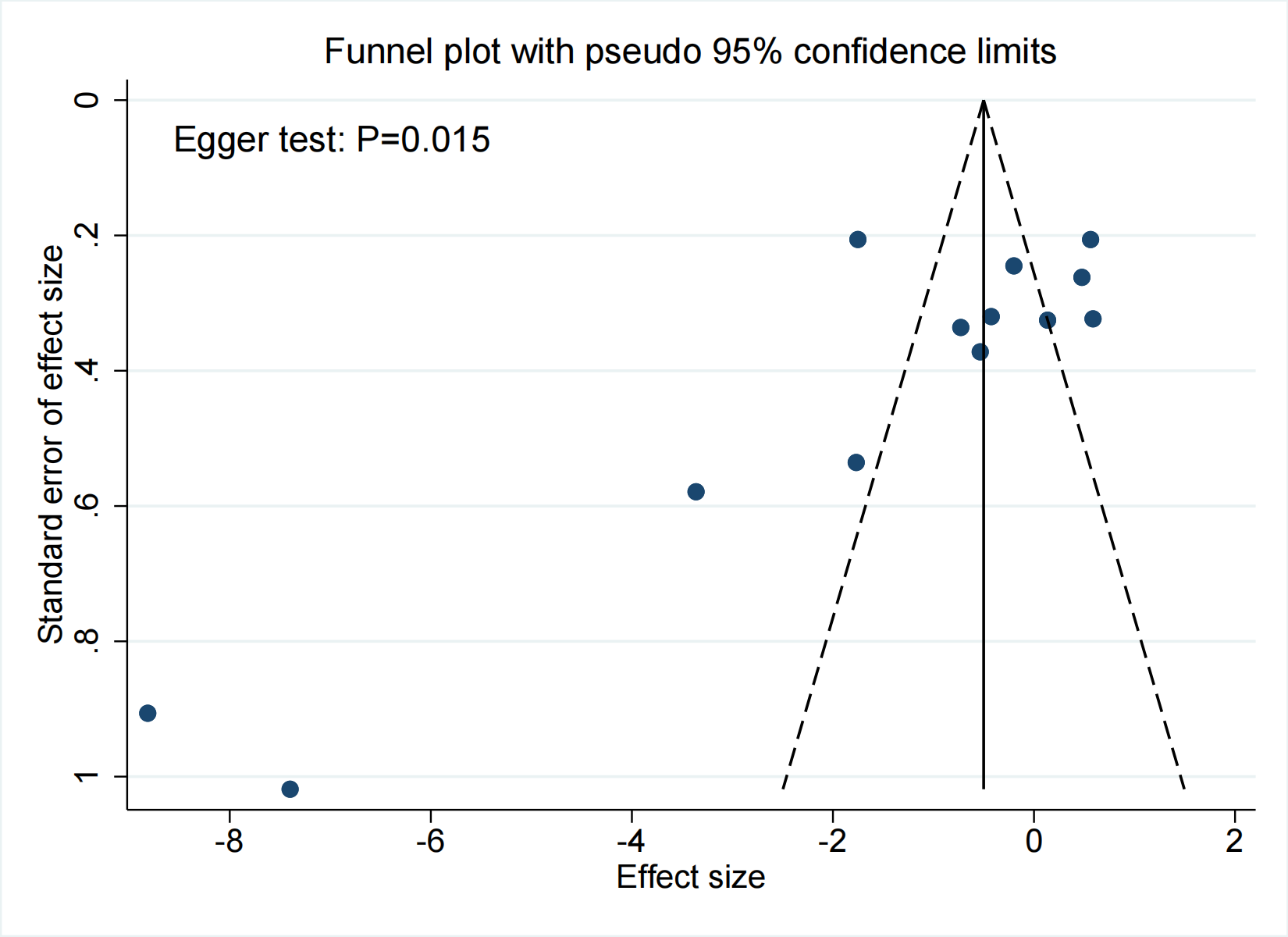


Figure 5.2 The funnel plot of PD. The result of Egger test showed the p=0.015.


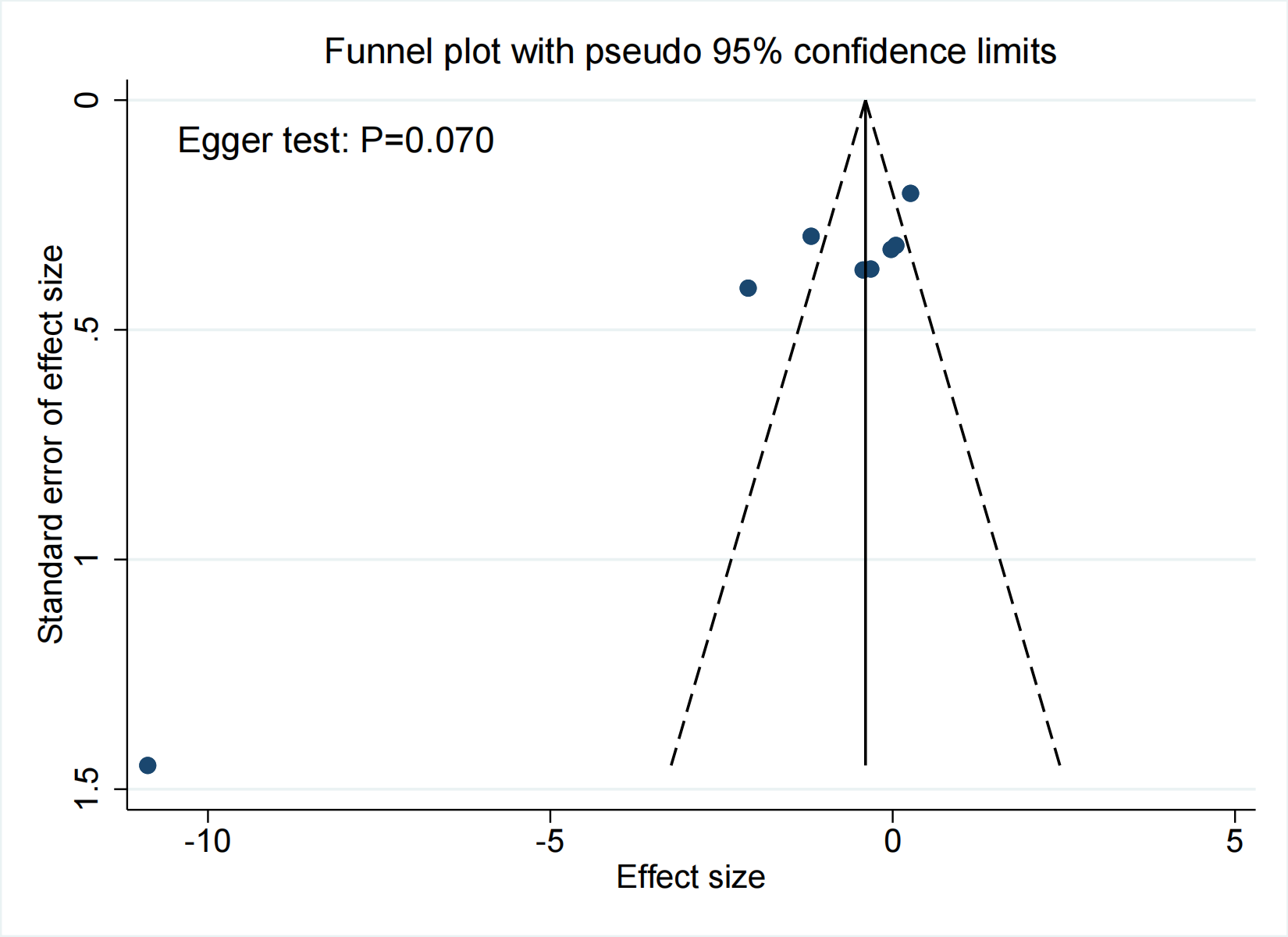


Figure 5.3 The funnel plot of PI. The result of Egger test showed the p=0.070.


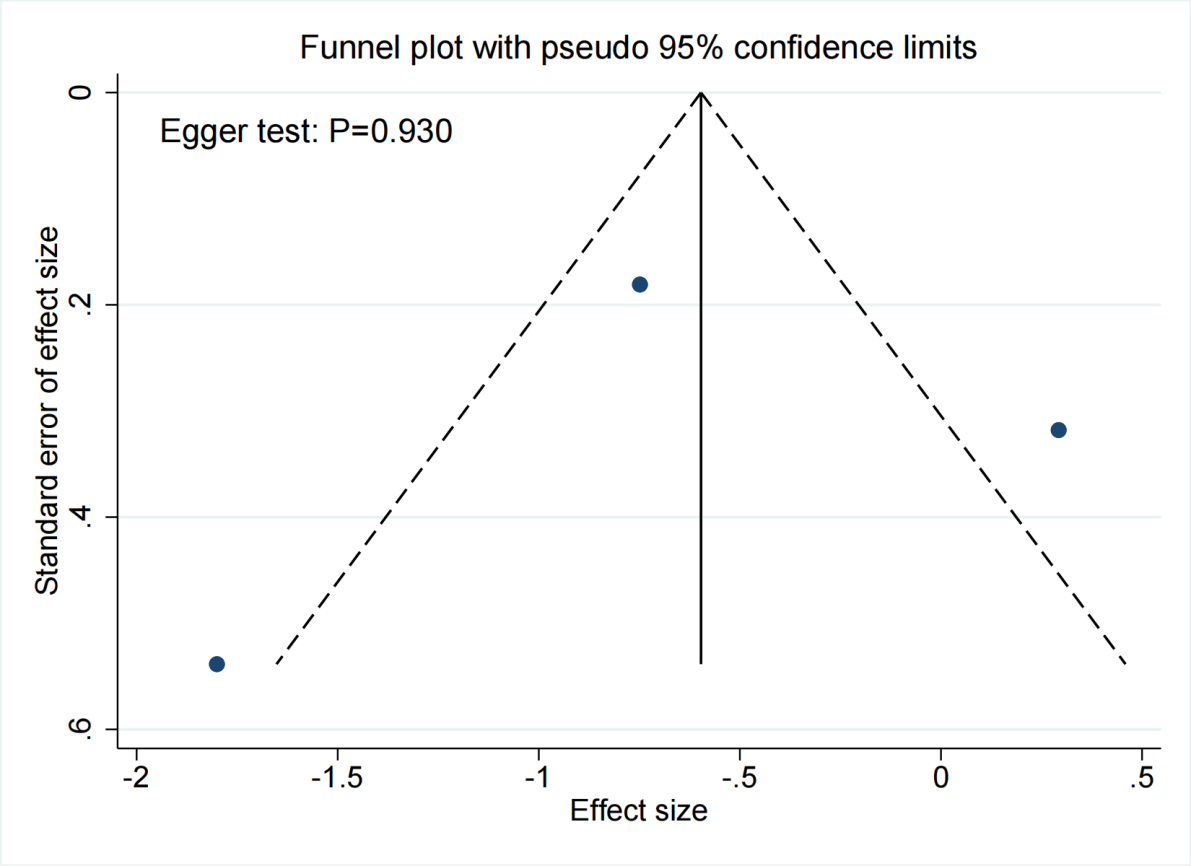


Figure 5.4 The funnel plot of CAL. The result of Egger test showed the p=0.930.


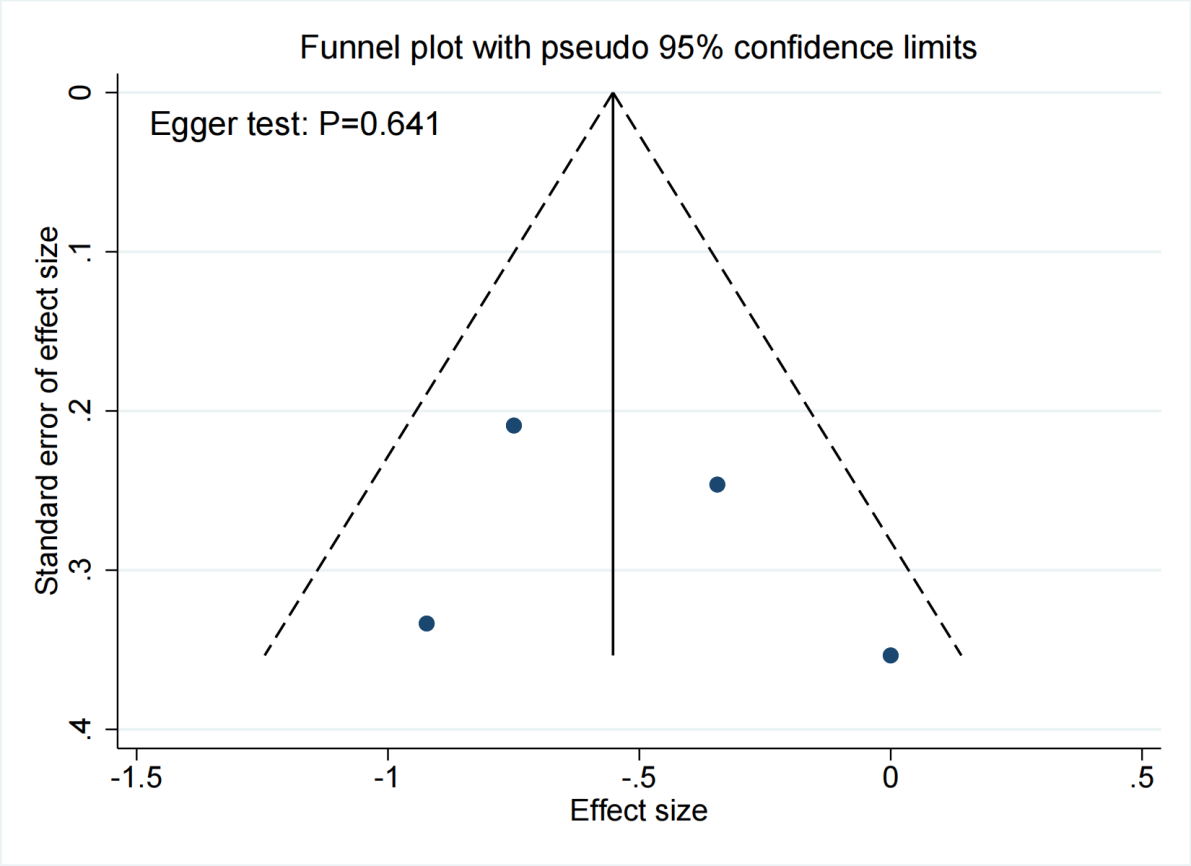


Figure 5.5 The funnel plot of CBL. The result of Egger test showed the p=0.641.


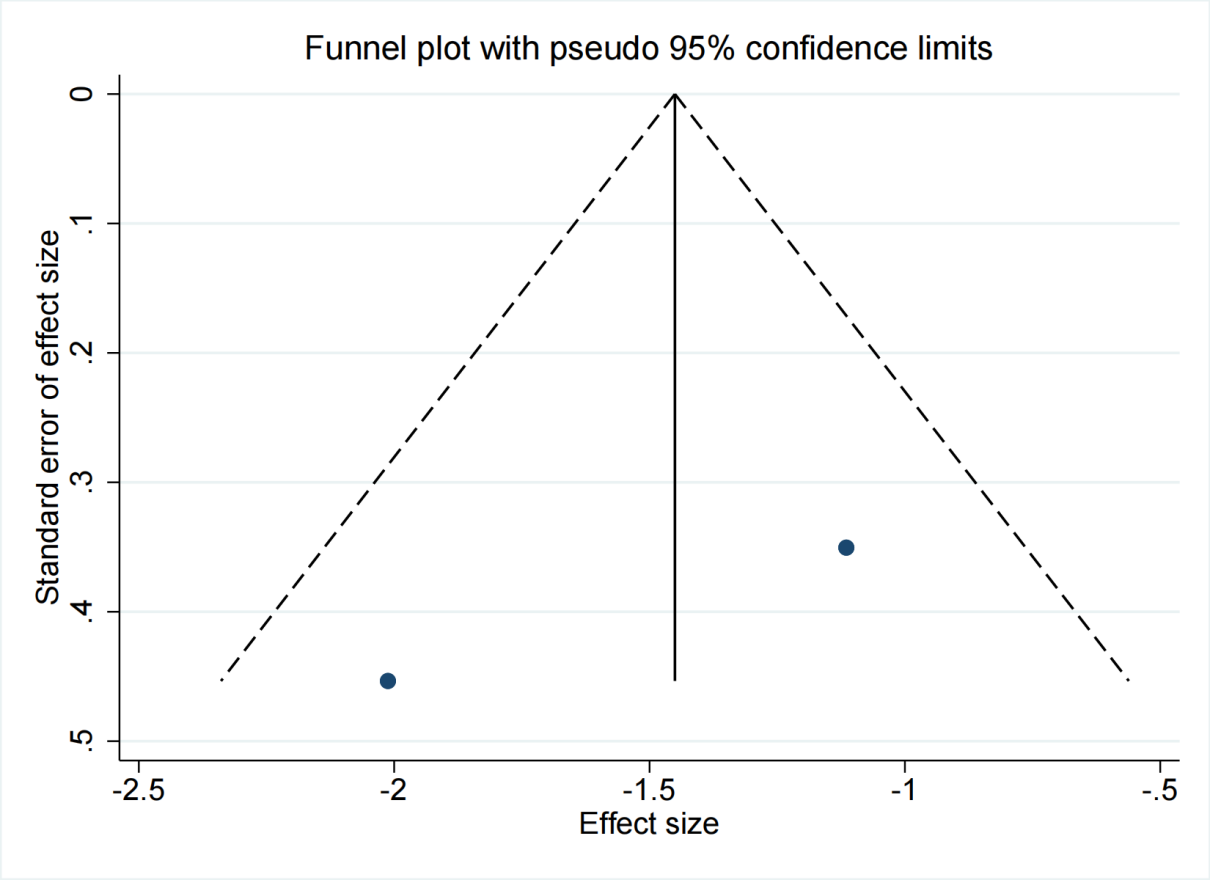


Figure 5.6 The funnel plot of BI.
